# Raspberry Shake-Based Rapid Structural Identification of Existing Buildings Subject to Earthquake Ground Motion: The Case Study of Bucharest

**DOI:** 10.3390/s22134787

**Published:** 2022-06-24

**Authors:** Ali Güney Özcebe, Alexandru Tiganescu, Ekin Ozer, Caterina Negulescu, Juan Jose Galiana-Merino, Enrico Tubaldi, Dragos Toma-Danila, Sergio Molina, Alireza Kharazian, Francesca Bozzoni, Barbara Borzi, Stefan Florin Balan

**Affiliations:** 1 European Centre for Training and Research in Earthquake Engineering (EUCENTRE), 27100 Pavia, Italy; ali.ozcebe@eucentre.it (A.G.Ö.); francesca.bozzoni@eucentre.it (F.B.); barbara.borzi@eucentre.it (B.B.); 2 National Institute for Earth Physics, Calugareni 12, 077125 Magurele, Romania; toma@infp.ro (D.T.-D.); sbalan@infp.ro (S.F.B.); 3 Department of Civil and Environmental Engineering, University of Strathclyde, Glasgow G1 1XQ, UK; ekin.ozer@ucd.ie (E.O.); enrico.tubaldi@strath.ac.uk (E.T.); 4 School of Civil Engineering, University College Dublin, D04V1W8 Dublin, Ireland; 5 French Geological Survey (BRGM), 45060 Orléans, France; c.negulescu@brgm.fr; 6 Department of Physics, Systems Engineering and Signal Theory, University of Alicante, Crta. San Vicente del Raspeig, s/n, 03080 Alicante, Spain; 7 University Institute of Physics Applied to Sciences and Technologies, University of Alicante, Crta. San Vicente del Raspeig, s/n, 03080 Alicante, Spain; 8 Department of Applied Physics, University of Alicante, Crta. San Vicente del Raspeig, s/n, 03080 Alicante, Spain; sergio.molina@ua.es; 9 Multidisciplinary Institute for Environmental Studies, University of Alicante, Crta. San Vicente del Raspeig, s/n, 03080 Alicante, Spain; alireza.kharazian@gcloud.ua.es

**Keywords:** Raspberry Shake 4D, modal identification, vibration-based structural health monitoring, rapid response to earthquakes (RRE), TURNkey project

## Abstract

The Internet of things concept empowered by low-cost sensor technologies and headless computers has upscaled the applicability of vibration monitoring systems in recent years. Raspberry Shake devices are among those systems, constituting a crowdsourcing framework and forming a worldwide seismic network of over a thousand nodes. While Raspberry Shake devices have been proven to densify seismograph arrays efficiently, their potential for structural health monitoring (SHM) is still unknown and is open to discovery. This paper presents recent findings from existing buildings located in Bucharest (Romania) equipped with Raspberry Shake 4D (RS4D) devices, whose signal recorded under multiple seismic events has been analyzed using different modal identification algorithms. The obtained results show that RS4D modules can capture the building vibration behavior despite the short-duration and low-amplitude excitation sources. Based on 15 RS4D device readings from five different multistorey buildings, the results do not indicate damage in terms of modal frequency decay. The findings of this research propose a baseline for future seismic events that can track the changes in vibration characteristics as a consequence of future strong earthquakes. In summary, this research presents multi-device, multi-testbed, and multi-algorithm evidence on the feasibility of RS4D modules as SHM instruments, which are yet to be explored in earthquake engineering.

## 1. Introduction

Vibration-based structural health monitoring (SHM) uses changes in dynamic characteristics of structures to detect, locate, quantify, and estimate the residual life-related consequences of damage reflected on the measurements [1,2]. Traditional SHM systems rely on heavy, bulky, high-precision, high-sensitivity, pricy, cabled, and centralized data acquisition standards. Within the last two decades, there has been an increasing trend of switching from cabled to wireless and centralized to distributed systems [3]. New SHM platforms are instrumented with smart sensors capable of self-sufficiency in terms of processing data, connecting to nodes, transferring signals, and operating the system [4]. While the communication and processing matters constitute the main effort behind smart sensors, new advancements in sensor technologies have radically reduced the cost per sensor node from four–five digits to three digits based on the latest advancements. Technologies such as micro-electro-mechanical systems (MEMS) enabled the production of cost-efficient accelerometers and other sensory devices contributing to the new age in the SHM discipline [5]. These advancements combined with the modern data science frontiers promote spatially dense, scalable, and affordable SHM solutions with a compromise on the merits of high-fidelity instrumentation [6].

MEMS technology has brought the sensor sizes to very small dimensions within millimeters. This has allowed the embedding of sensors within many industrial and consumer-grade devices (e.g., smartphones) and deploying next-generation sensing modules empowered by low-cost programmable boards (e.g., Arduino, Raspberry Pi). Smartphones, for example, provide vibration measurement alternatives that could be used for experimental modal analysis with support from heterogeneous sensory and crowd-enabled environments [7]. In line with the smart sensor advancements, Internet of things (IoT) protocols have assured everyday connectivity between cloud and operating sensor nodes [8,9]. IoT nodes have found deployable forms with the contribution of low-cost programmable boards and MEMS sensors for many engineering applications, for example, SHM as well as engineering seismology [10]. One main initiative in seismology is the advent of cost-efficient and crowdsourcing-based seismometer networks, and Raspberry-Pi-based Raspberry Shake products have risen as one of the most prominent solutions integrating different sensors within the same module (e.g., multiaxial accelerometers, geophones, and on-board processing, computation, and communication) [11].

While Raspberry Shake devices spread around the world rapidly to form a new generation seismic network, their capabilities as SHM instrumentation for modal identification and damage detection are yet to be discovered [12]. If Raspberry Shake devices can serve for dynamic identification of structural systems before and after extreme events such as earthquakes, one can retrieve sensor-driven estimations on the condition of the civil infrastructure and account for the extent of the damage in massive scales covered by the network. While high operational vibrations in bridge infrastructure would trivially ensure that low-quality MEMS data can still capture structural characteristics due to the high signal-to-noise ratio [13], the feasibility of building infrastructure is still unclear due to the lack of natural excitation sources in the majority of the stock. The buildings experience low-amplitude vibrations under the ambient conditions, and thus have the risk of being below the required signal ranges and having measurements masked by noise. Earthquakes of sufficiently high intensity, however, can generate the signal level above the essential range and thus enable the capture of the building dynamic behavior through Raspberry Shake nodes. In this research, the authors explore the dynamic identification of six buildings, based on low-amplitude seismic events.

As part of the TURNkey project [14], the authors have developed a knowledge-adaptive framework to relate modal parameter changes to building damage, expressed in terms of the European Macroseismic Scale damage grades (EMS98) [15]. In Ozer et al. [16], the authors identified alternative damage-assessment approaches empowered by empirical frequency-damage relationships [17] as well as analytical formulations based on an effective stiffness concept [18,19]. In all these approaches, period elongation, identified through various modal identification algorithms, serves as a proxy for the damage experienced by the building under the earthquake loading. While independently formulated in earthquake engineering, such a formulation is in line with the standards set by Rytter [20] covering the detection and quantification phases of SHM-based damage detection. Quantifying the level of damage can also be based on alternative approaches other than vibration-based frequency decay, for example, monitoring of engineering demand parameters and associated exceedance criteria under earthquake excitation [21]. 

As introduced earlier, due to the high cost of sensors, SHM is often applied only to critical civil infrastructures, such as long-span bridges [22,23] and supertall buildings [24,25]. On the other hand, there is an increasing demand for low-cost sensors for earthquake early warning (EEW) (e.g., [26]) and rapid response to earthquakes (RRE). With a small economic burden, relatively large networks could be constructed, as in the case of the TURNkey network [27,28,29,30]. Low-cost sensors (e.g., MEMS) have proven useful and provided promising results also for small local earthquake detection (e.g., [31]) and initial ground-motion assessment (e.g., [32]). However, the reliability and performance of the Raspberry Shake sensors when used for SHM purposes have not been extensively studied yet. The present paper aims to analyze their potential use for the dynamic identification of structures, using data from low-magnitude Vrancea (Romania) seismic events.

As expressed, structural characterization through SHM remains an open research field. To engage the knowledge-adaptive framework described above with actual testbeds, this study presents real-world structures from Bucharest (Romania), which are instrumented with 15 Raspberry Shake 4D (RS4D) multi-sensor units. Within the framework of the TURNkey project, continuous real-time structural monitoring of five different structures has been achieved through low-cost RS4D sensors in Bucharest, being exposed to a high level of seismic hazard, governed mainly by the Vrancea intermediate-depth seismic source [33,34,35,36]. 

The SHM research in Romania was initiated in the 1960s at URBAN-INCERC [37,38]. By the time of the 1977 earthquake, there were two structures with permanent seismic instrumentation and an extensive ambient vibrations measurement campaign was conducted to determine the dynamic parameters of 47 residential buildings in Bucharest, with various structural systems and numbers of stories ranging from 8 to 18. The aim was to compute reference values of fundamental periods to be used in case of a strong earthquake. Post-event studies suggested indeed the correlation of the damage level with the elongation percentage of the fundamental period of the structures, concerning the undamaged reference values. Nowadays, the interest in SHM systems in Romania is increasing. Several private companies are starting to perform this operation and the main academic and research institutes are starting to collaborate to harmonize the work in the field and boost the interest in this topic.

In Romania, the National Institute for Earth Physics (INFP) started instrumenting structures within research projects in the late 1990s, and since 2011 they have extended the network. Currently, INFP is in charge of monitoring 10 buildings in Romania, a key moment being achieved in 2020 when the TURNkey network comprising the 15 RS4D multi-sensor units was deployed in Bucharest. A focused review of the seismic instrumentation of structures in Romania, evolution, recent progress, and perspectives is presented in [39].

Recent earthquakes occurring in the Vrancea intermediate-depth seismic region generated valuable datasets comprising real earthquake signals captured by RS4D devices. In this paper, the SHM methodologies presented in [16] are tested on RS4D data and comparisons are also made with the signals from high-fidelity broadband sensors installed in a benchmark structure. Even though no damage was identified due to the weak nature of the seismic signals, this paper provides evidence of acquiring modal characteristics of the buildings excited by the earthquakes. The rest of the paper is organized as follows: Section 2 introduces the RS4D modules and instrumentation deployed on multiple buildings situated in the testbed region; Section 3 investigates the dataset developed during the latest seismic activities in the region recorded from multiple buildings via RS4Ds; Section 4 briefs the identification techniques capturing the modal characteristics of building infrastructure; and finally, Section 5 presents the conclusive findings and further research directions.

## 2. Instrumentation and Specification

**Figure 1:**
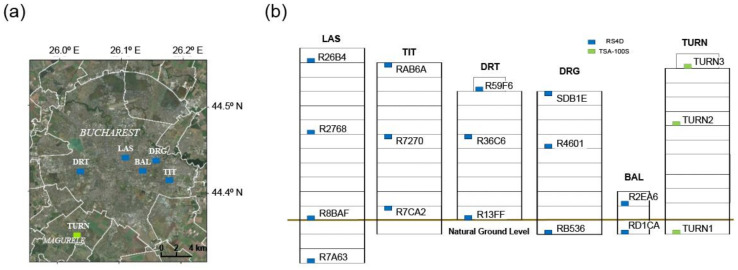
Information on the instrumented buildings in Bucharest within the TURNkey context. (**a**) Map of Bucharest with the location of the six instrumented buildings within the TURNkey project and (**b**) instrumentation scheme for each building and sensor type (RS4D—blue; TSA-100S—green).

In 2020, 15 TURNkey multi-sensor units (equipped with 15 RS4D sensors and 5 GNSS sensors) were deployed in five buildings, identified as BAL, DRG, DRT, LAS, and TIT. These buildings represent three different generations of earthquake-resistant design codes and were chosen to understand the effect of earthquakes on structural typologies exhibiting different vulnerabilities. In addition, a sixth building (labelled as TURN) was added afterwards as a case study for the project, since it was instrumented for a long time (since December 2013) with high-fidelity accelerometers (TSA-100S tri-axial accelerometers and a 24-bit Digitizer, iDAS). Figure 1 illustrates the location of the monitored buildings and the accelerometers within the buildings, whereas Table 1 details the main characteristics of the instrumented structures.

The first official Romanian seismic design code was introduced in 1963 [40]. Before that, based on the experience of the 1940 earthquake that hit Romania, recommendations were only made to consider a seismic coefficient of 5% in the design. Successive improvements to the Romanian seismic design codes have been made after the 1977 earthquake, with the advancement of research in the field and, most importantly, with recorded data that served as a basis for the updated seismic design spectra. The modern Romanian seismic design codes are in line with the recommendation of the Eurocode 8 [41] and were enforced in 2006 [42] and 2013 [43].

### 2.1. RS4D Sensors

**Figure 2:**
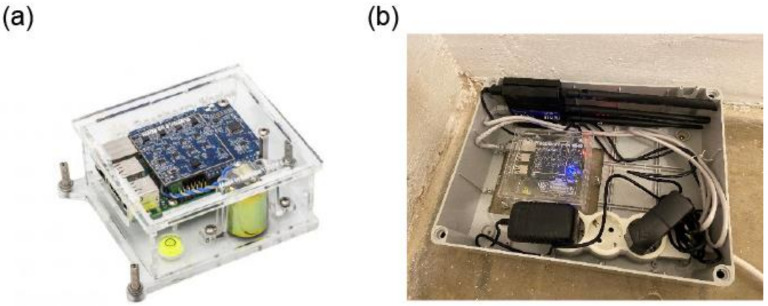
(**a**) Raspberry Shake 4D sensor (three-component MEMS accelerometer and one vertical geophone) and (**b**) the installation setup for Bucharest, with power supply, protection and GPRS router.

RS4D (Figure 2) is a well-rounded, strong motion seismograph. Equipped with multiple sensors, more data options and a wider range of applications are possible, also including the recording of the earthquake ground motion (EGM). RS4D has three orthogonally positioned MEMS, where tri-directional accelerometers are built into the board and a professional-grade vertical seismometer has a geophone, as well. Various types of signals having different origins (natural or anthropogenic), either transient or continuous small-amplitude ground vibrations are often referred to as seismic noise. It is well known that the COVID-19 lockdown measures on human and industrial activities have reduced the anthropogenic seismic noise at the global level, especially for high-frequency vibrations [44], including in Romania and Bucharest [45]. The performance of the RS4D in the context of monitoring the propagation of seismic waves was assessed with laboratory tests and field observations by [46]. The authors concluded that the largest drawback of the RS4Ds is the high self-noise of the instruments, compared to the high-fidelity, broadband sensors typically used in seismic networks. Due to the high level of self-noise, the authors concluded that MEMS accelerometers are not suitable for studies using ambient vibrations or teleseismic events, but they are much better suited for the characterization of local (<10 km) events of magnitude M > 2.5 or regional (<100 km) events of M > 5.0. However, considering the ease of installation, the relatively low-cost and the real-time data transmission, the RS4D sensors can be an ideal candidate for the densification of the seismic networks for local and regional events.

**Figure 3:**
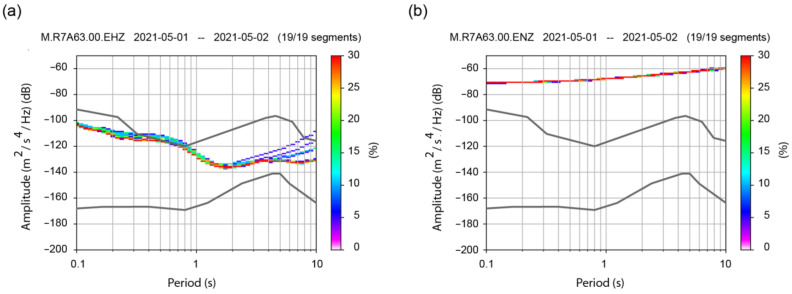
The noise level (power spectral density functions) of one RS4D sensor (LAS 3rd basement) during the quietest period of recordings (five-hour window), with respect to the NLNM and the NHNM (black lines) for the (**a**) velocity channel and (**b**) acceleration channel.

Similar observations on instrumental self-noise are valid also for the sensors installed on the monitored buildings of the TURNkey project. The noise level was tested by using the McNamara and Buland methodology [47], based on data recorded during the quietest period since the deployment of the instruments. Five hours during a weekend night were selected, for the station located at the LAS 3rd basement. Being an office building, its activity during the weekend was reduced. In addition, the 1 May 2021 weekend was a National Holiday in Romania, thus many residents left Bucharest, contributing to further reductions in the city’s ambient vibrations. With the reference to the standard low noise model (NLNM) and high noise model (NHNM) for seismic stations [48] plotted with black lines, Figure 3 shows the probabilistic power spectral density (PPSD) plots computed by using the Obspy routines [49], which are quite consistent with the ones present in the RS4D technical specification document [50]. The five-hour interval was split into 19 data segments for which PPSD estimates were computed. Even though the vertical geophone is sensitive due to its low value of self-noise (Figure 3a), during a strong EGM the geophone velocity sensor can be “saturated” or exceed the instrument’s measurement limit. The integrated MEMS accelerometer should remain in scale during strong EGM, with the cost of a higher level of self-noise, as presented in Figure 3b. While for the velocity channel (Figure 3a) all the analyzed segments fall below or slightly exceeds (for periods from 0.4–0.8 s) the NHNM limit, for the acceleration channel (Figure 3b) the upper limit is exceeded on the entire period range of interest (0.1–10 s), thus suggesting that the acceleration recordings are unreliable for ambient vibration analysis (as highlighted also in the next section) or small-amplitude Vrancea seismic events (M < 4.5). However, the two types of sensors are complementary and proved to work well together.

**Figure 4:**
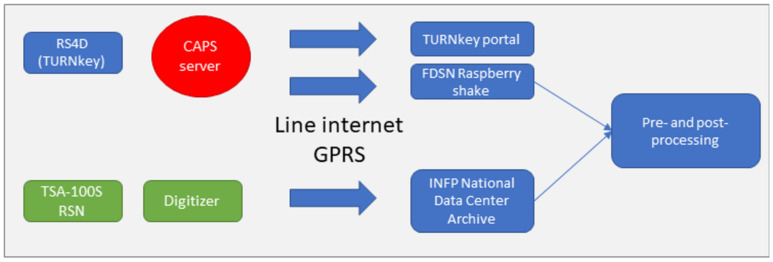
Data-flow transmission scheme used within the TURNkey project for Bucharest testbed.

All stations were configured to forward data to the TURNkey platform server hosted at Gempa. The Common Acquisition Protocol Server (CAPS) was developed to fulfil the need to transfer multi-sensor data from the station to the data center (https://www.gempa.de/products/caps/) (accessed on 30 May 2022). In addition, the CAPS data compression module was implemented in the units and the data stream was rerouted to the Gempa CAPS system (Figure 4).

### 2.2. Broadband Sensors

**Figure 5:**
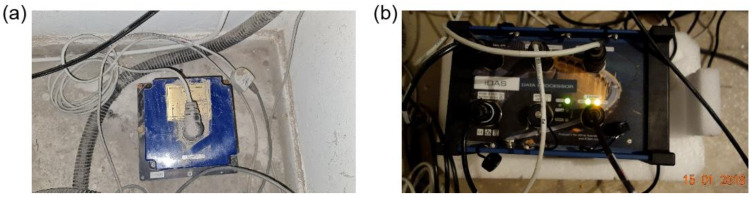
(**a**) TSA-100S sensor and (**b**) iDAS Digitizer as installed on the TURN building.

The Institute of Atomic Physics building (TURN) is located in Magurele, close to Bucharest. The building was completed in 1974, it was partially damaged by the 1977 earthquake, and it was retrofitted afterwards, in the 1990s. The monitoring system consists of three accelerometers installed in the basement, 6th floor and 10th floor. The three TSA-100S tri-axial accelerometers and a 24-bit Digitizer (iDAS) were installed in December 2013 and all the data are transmitted in real-time to the INFP’s National Data Center, for the purposes of archiving and post-processing (Figure 5).

## 3. Seismicity and Dataset

Romania is an active seismic country and Bucharest is one of the most earthquake-endangered capitals in Europe [51,52], threatened mostly by the earthquakes in the Vrancea intermediate-depth seismic source [33,53]. In every century, one to five strong earthquakes (M_W_ > 7.0) have occurred in Romania [33], with the most recent destructive ones being in 1940 and 1977. In 1977, due to the large values of spectral accelerations for long periods in Bucharest [54], 32 (most of them high-rise) buildings were completely or partially damaged, causing the deaths of 1424 people and 7598 injuries [54,55,56], which ranks the 1977 event as the most destructive earthquake in the country. 

Bucharest is located in the central part of the Moesian platform, in the so-called Romanian Plain, at around 165 km away from the Vrancea intermediate-depth seismic source [57,58,59,60,61]. The elevation lies between 60 and 95 m above sea level, with elevation contour lines generally oriented NE-SW, roughly perpendicular to the direction of the main rivers going across the city, which are the Dambovita and Colentina [58]. The near-surface geology consists mainly of Quaternary alluvial deposits (total thickness varies from 200 m in the South to 300 m in the north of the city) [62], consisting of the following lithological layers: (a) loess-like deposits and the alluvia of the Dambovita and Colentina rivers; (b) Colentina gravels and sands; (c) intermediate-clay deposits; (d) Mostistea sands; (e) Marl complex; and (f) Fratesti layers [58,59]. Based onV_S30_ borehole data, the entire Bucharest territory corresponds to soil type C according to the Eurocode 8 classification [41,59]. However, it was proven that in the case of Bucharest, laying on deep sedimentary layers, the V_S30_ classification is inadequate [58]. The amplification peak correlated in the literature with the base of the Quaternary deposits [63] was attributed to the engineering bedrock [60] and it is in the range of 1.1–1.6 s (e.g., [61,63,64,65]). This peak was predominant for the 1977 Vrancea earthquake and it is of great interest to the Romanian earthquake engineering community. The plateau of the constant maximum design spectral acceleration is extended up to 1.6 s (T_C_ = 1.6 s) in the Romanian seismic design code [43] for Bucharest. A list of the most recent earthquakes between June 2020 and June 2021, starting with the multi-sensor deployment campaign within TURNkey, is presented in Table 2, according to the Romanian Earthquake Catalogue—ROMPLUS [66]. The largest earthquakes that were recorded since the TURNkey network was deployed occurred on 9 April and 25 May 2021 (bold).

**Figure 6:**
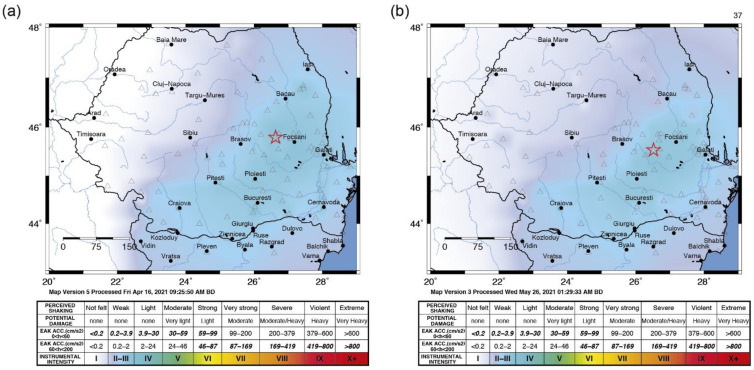
INFP generated shake maps in terms of intensity for (**a**) the 9 April 2021 earthquake and (**b**) the 25 May 2021 earthquake. The star denotes the epicenter.

The 9 April 2021 earthquake had occurred in the Vrancea intermediate-depth seismic zone, at 21:36:47 (local time) and a depth of 79 km. The earthquake epicenter was located 156 km north of Bucharest and its maximum epicentral intensity was III on the modified Mercalli intensity (MMI) scale. The earthquake was felt on the Romanian territory and the maximum recorded acceleration by the Romanian National Seismic Network was 4.4 cm/s^2^, close to Piatra-Neamt, in the northern part of the Vrancea seismic source. The 25 May earthquake occurred in the Vrancea intermediate-depth seismic zone, at 00:30:37 (26 May local time) and a depth of 131 km. The earthquake was located 126 km north of Bucharest and its maximum epicentral intensity was IV MMI. The earthquake was felt in the Romanian territory and the maximum recorded acceleration by the Romanian National Seismic Network was 6.6 cm/s^2^, in Braila County, the eastern part with respect to the Vrancea seismic source. The intensity distribution (ShakeMap) for the two earthquakes is presented in Figure 6. Even though the difference in magnitude of the earthquakes was only 0.1 and the local site conditions are the same for both events, the signal recorded by the seismic stations in buildings was also influenced by the different source properties (depth, magnitude, focal mechanism) and propagation distance. The focal mechanism of the 25 May earthquake indicates reverse faulting, with compression axis orientation NW–SE and extension axis almost vertical, while the 9 April earthquake mechanism also indicated reverse faulting, but with an important strike-slip component.

**Figure 7:**
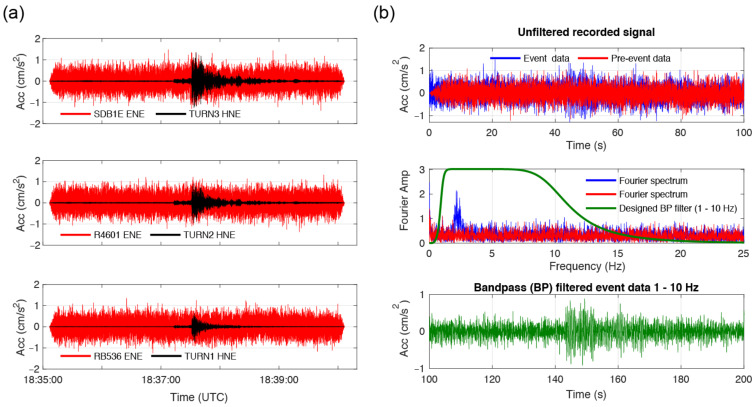
(**a**) Comparison of the east–west acceleration time-histories of the RS4D sensors in the DRG building and of the TSA-100S sensors in the TURN building during 9 April 2021 event. (**b**) RS4D acceleration data analysis (SDB1E sensor) in terms of 100 s pre-event (red line) and during event 100 s (blue line) recordings and associated Fourier spectra of the signals. The green line in the bottom plot represents the 1–10 Hz filtered event signal in the time domain and the corresponding designed bandpass filter in the frequency domain.

Figure 7a illustrates the comparison of the recordings of the RS4D sensors at the DRG building and the recordings of the TSA-100S sensors at the TURN building during the 9 April 2021 event (300 s window, including pre-event noise recordings). In Figure 7b, first, the data recorded on the roof level (SDB1E ENE) is compared with and without the presence of the earthquake, then, the Fourier spectra of the noise and earthquake signal are overlapped with the filter used, and finally, the seismic signal is further highlighted through use of the band-pass filter previously designed.

It can be observed that the high value of the instrument self-noise masks the seismic signal in the acceleration recordings of the RS4D sensors (i.e., too low signal over noise (S/N) ratio). The root mean square (RMS) of the pre-event top RS4D recording (100 s) is 0.3 cm/s^2^. This is consistent with the RMS noise level (0.0003 g) provided by the manufacturer of the MEMS accelerometer [50] and confirms that no pre-event modal identification of the structure can be achieved based on the RS4D recordings. On the other hand, for the same pre-event time interval, the RMS of the TSA-100S recording (TURN3) is about 0.01 cm/s^2^. However, this value for the TURN building is influenced by other low-amplitude vibrations in the building that exceed the self-noise of the instrument.

## 4. SHM Methodologies

### 4.1. Output-Only Methods

The output-only modal identification methods used in this study consist of: (a) frequency domain decomposition (FDD), (b) continuous wavelet transform (CWT), and (c) short-time Fourier transform with wavelet pre-filtering (STFT-WF). Details and their use in this particular work are explained in the following subsections.

#### 4.1.1. Frequency Domain Decomposition (FDD)

Frequency domain decomposition (FDD) is one of the benchmark techniques used to identify the modal frequency of the testbed buildings studied in this paper. Brincker et al. [67] developed the output-only technique which operates with the condition that the input motion is white noise, and FDD is capable of producing modal frequencies and mode shapes of the structure without input knowledge. FDD uses multi-output data to develop power spectral density (PSD) matrices as a function of frequency and uses singular value decomposition (SVD) to identify eigenvalues and eigenvectors of each PSD matrix per given frequency. High eigenvalues at certain frequencies indicate potential modal frequencies and the eigenvectors correspond to mode shape knowledge paired with that frequency. The mathematical expression for this operation is given in Equation (1).

(1) $$S_{yy}\left(w\right)=U\left(w\right)\cdot \Sigma \left(w\right)\cdot U^{H}\left(w\right)$$

where $S_{yy}\left(w\right)$ is the PSD matrix of the response vector *y*, $U\left(w\right)$ is the unitary matrix of the singular vectors, $\Sigma \left(w\right)$ is the diagonal matrix of the singular values, and *H* denotes the complex conjugate transpose operator. The $\Sigma \left(w\right)$ component of $S_{yy}\left(w\right)$ contains information regarding frequencies containing high eigenvalues, and other components provide eigenvector-related mode shape information. Further information concerning the details of the technique can be found in [67].

The technique is not ideal when used under non-white noise excitation, therefore, it is deemed more appropriate to use a technique that incorporates input motion as well as outputs (for example, stochastic subspace identification). However, these techniques need additional processing such as stabilization diagrams [68] and hierarchical clustering [69] when subject to high noise, and they can be computationally expensive, necessitating the use of computationally efficient single-output techniques as pursued in this work. One can note that singular value spectrums alone may be insufficient to determine modal characteristics but repeatedly identified frequencies further solidify the conclusion that the output vibration mode is not spurious. Furthermore, the presence of the remaining methods further solidifies the outputs of FDD, as well. In such a combined manner, inter- and intra-verifications, and non-structural identifications of FDD may be further eliminated.

#### 4.1.2. Short-Time Fourier Transform with Wavelet Pre-Filtering (STFT-WF)

The discrete wavelet transform (DWT) can be roughly explained as a sub-band coding scheme [70], where the signal is iteratively split into two frequency bands (low-pass and high-pass) by applying two quadrature filters plus down-sampling. These filters are known as scaling and wavelet filters, respectively, and are derived from the base function known as mother wavelet. The coefficients obtained from low-pass filters (approximation coefficients) represent the approximation of the signal at different scales. On the other side, high-pass filters provide the wavelet coefficients, which represent the details of the signal at different scales or resolutions. More details of the discrete wavelet analysis can be found in Daubechies [71], Wickerhauser [72], and Strang and Nguyen [73].

Once the signal is decomposed into wavelet and approximation coefficients, the contribution of one or several of these coefficients to the original signal can be analyzed by applying zero for the rest of the coefficients and then reconstructing the signal through the inverse transform. Thus, a wavelet band-pass filter can be accomplished (e.g., [74,75]), significatively improving the quality of the signal by the selection of the frequencies of interest.

In the present work, for the sampling frequency used and the expected frequencies, the recorded signals have been decomposed into six levels or scales using the basis Daubechies 12 as mother wavelet. After that, the approximation coefficients of the full scale were selected to discard the high frequencies contained in the signal.

After the wavelet pre–filtering (WF), the short-time Fourier transform (STFT) is applied to the resulting signal. It is a Fourier-related transform where the recorded signal is divided into shorter intervals of equal length, and then the Fourier transform is applied separately in each of the intervals. With this approach, not only can the fundamental frequency be estimated but also its variation with time can be observed, especially from intervals of only seismic noise, ground motion, or coda recordings.

In order to minimize the discontinuity effects in the borders of the time windows or intervals, it is recommended to multiply each interval by a window function that smooths the selected segment [76]. In our case, 5% cosine tapering has been used. After that, the Fourier transform is applied, and the corresponding spectrum is obtained for each time window. Additionally, a Konno–Ohmachi filter [77], with constant b = 40, is applied in the frequency domain in order to smooth the previously calculated spectra.

The length of the time windows is inversely proportional to the minimum frequency to be investigated (Rayleigh frequency) [78,79]. Nevertheless, for a reliable measurement, it is recommended that the study frequency be at least ten times above the minimum frequency, which means that at least 10 cycles of the signal are analyzed in each window [80]. In the present study, 30 s windows have been used which allows retrieving reliable fundamental frequencies above 0.3 Hz. The fundamental frequency is estimated for every analyzed time window. Thus, the mean fundamental frequency and its corresponding standard deviation are provided as a final result.

#### 4.1.3. Continuous Wavelet Transform (CWT)

Wavelet analysis decomposes a time series into frequency and time domains using a set of window functions (named “wavelets”) that have compact support in time (i.e., decays to zero quickly) and are band-limited in the frequency domain. The basic idea is to find a function *ψ*(*t*), which can generate a basis for the entire domain of function *x*(*t*). Wavelet analysis consists of different forms of transformations, such as the continuous wavelet transformation (e.g., [81,82]), the discrete wavelet transformation (e.g., [83]), and the wavelet packet transformation (e.g., [84]). In this study we are using a CWT that decomposes a function *x*(*t*) into the frequency-time domain as defined in Equation (2):

(2) $$W_{\left(a,b\right)}=\frac{1}{\sqrt{a}}\int_{-a}^{+a}x\left(t\right)\cdot \psi^{*}\left(\frac{t-b}{a}\right)dt$$

where ψ*(*t*) is the complex conjugate of *ψ*(*t*), *b* is the parameter localizing the wavelet function in the time domain, and W(*a,b*) is the CWT coefficient that represents the measure of the similarity between the function *x*(*t*) and the wavelet at the time *b* and the wavelet scale *a*. 

In this study, the complex Morlet wavelet is used. This wavelet is expressed by *f*: Fourier frequency, *fc*: wavelet central frequency, and *fb*: bandwidth parameter. The conversion between *f* and *a* (wavelet scale value) is a function of the *fc* and *fs* (the sampling frequency). The choice of these parameters is crucial in order not to miss the frequencies of the response. In the calculations, the values chosen are *fb* = 1, *fc* = 3, and a proper *a*-value for the frequencies of lowest and highest frequencies of interest of 20 and 250 Hz.

The method ideally relies on the localization of the energy content of the tail of the record under consideration. This application provides reliable results when the system provides high S/N ratios. For the details of the method, readers may refer to (e.g., [85,86]). It is noted that the accuracy of this approach has been recently benchmarked with a series of shake table experiments [16].

For the noisy systems (such as RS4D instruments), in this work, a walk-around solution is provided by considering the entire recording to locate the fundamental vibration frequency in the frequency space.

### 4.2. Input–Output Methods

Among the various input-output methods available in the literature, only the Stockwell transformation [87,88] based amplification function (ST-AF) approach is used. As shown and benchmarked in [16], the concept of the method is quite engineering-based. The ratio of the Stockwell transformations of the roof signal over the base one is calculated for all temporal instants as shown in Equation (3):

(3) $${\mathrm{AF}}_{\mathrm{ST}}=\frac{\left|\mathrm{ST}\left(acc_{r}\right)\right|}{\left|\mathrm{ST}\left(acc_{b}\right)\right|}$$

where AF_ST_ is the amplification function matrix in the time (t) and frequency (f) domain, ST is the function that carries out Stockwell transformation, *acc_r_* is the recorded acceleration at roof level, and *acc_b_* is the recorded acceleration at the base level.

The method seeks the frequency *(f)* at which the maximum amplification is observed in the time domain (t) once the amplification function matrix is calculated in terms of the *t-f* couple. In its raw version, the amplification function may have sparsely spaced singularities, especially when the instantaneous frequency content of the denominator approaches zero. Due to this reason, a parameter called *max_amp*, ranging from 2 to 15 can be used to cap the amplification function. When the *max_amp* is set low, a broad range of frequencies could be identified with having the amplification function equal to *max_amp*. In such cases, the instantaneous fundamental frequency is set as the mean of the maximum and minimum frequencies having *max_amp*. In this respect, for noisy systems (such as the combination of RS4D sensors and low-amplitude seismic vibrations), *max_amp* helps increase the precision of the inverted frequencies with the cost of losing the frequency resolution. For less noisy systems, higher *max_amp* values are used so that the unwanted effects become invisible.

For noisy systems, in which the signal becomes distinguishable from the noise during the shaking time (e.g., recording of RS4D sensors during weak shaking), the decision of “where to look at” becomes important in locating the instantaneous fundamental frequencies. To address this issue, following a preliminary set of analyses, three parameters are assigned: *f_int*, *t_int*, and *perc_tol*. Among these, *f_int* defines the interval in which the first vibration frequency exists (e.g., for BAL: 4 to 7 Hz, for the others: 0.5 to 3.0–3.5 Hz) which is predictable from empirical equations, *t_int* defines the time interval where the earthquake motion is the most intense, and *perc_tol* defines the tolerance controlling the minimum permissible instantaneous Stockwell transformation amplitude at the roof level (e.g., 50%).

Table 3 summarizes the modal identification methods considered in this study together with their main features.

## 5. Analyses

The analyses were carried out to (a) show the representative functionality of the methods described in the previous section through a benchmarking analysis of different SHM techniques by using the high-fidelity TSA-100S installed on the TURN building, (b) document a case study in which visual presentations of the method outputs when a set of RS4D instruments are used (e.g., shown for LAS), (c) provide repeatability analyses for selected two buildings instrumented with RS4D sensors (e.g., DRG and BAL) through the use of two distinct datasets belonging to two seismic events, and (d) provide a comparison of identified frequencies obtained in all the cases.

### 5.1. Comparison of the Methods Considering the Recordings from the High-Fidelity Sensors Installed in the TURN Building

**Figure 8:**
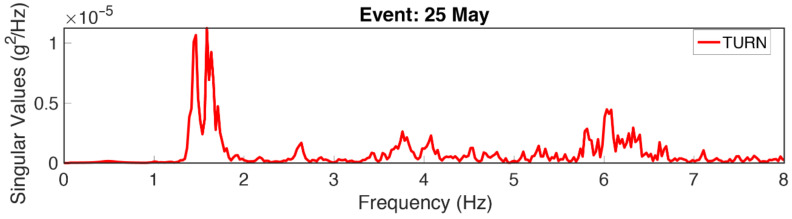
SVD as the output of FDD along the *x-direction* through the use of the dataset of the 25 May event recorded on TURN.

Figure 8 presents the SVD of the TURN building using the dataset of the 25 May event. Although the use of EGM data may potentially impose inaccuracies in the period identification due to the inclusion of the input frequency content, in this particular case the identified 1.6 Hz is far away from the incoming frequency content, as revealed by the application of the ST-AF method.

**Figure 9:**
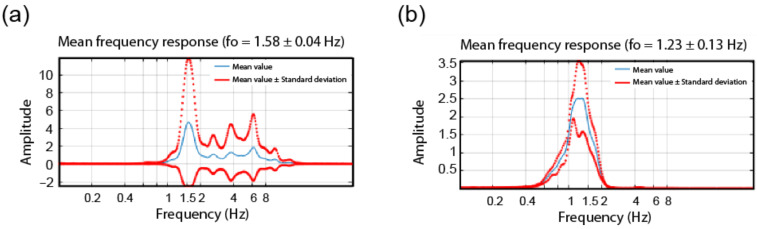
FADs as the output of STFT-WF technique along (**a**) *x* and (**b**) *y* directions.

Figure 9 presents the STFT-WF processing of the TURN building by using the dataset of the 25 May event in terms of FADs. Due to the presence of a high S/N ratio, no wavelet pre-filtering is needed. In both directions, natural vibration frequencies are found to be located at about 1.58 Hz.

**Figure 10:**
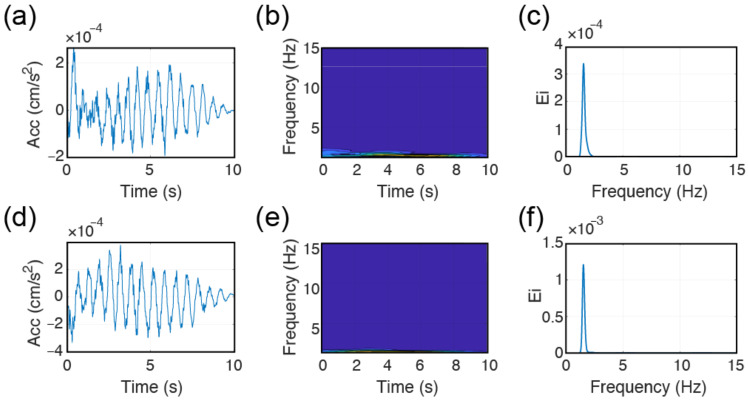
Results of CWT processing results: (**a**,**d**) acceleration-time history of the tail of the roof; (**b**,**e**) t–f–acceleration response of the roof data; (**c**,**f**) EFDs. (**a**–**c**) for *x* and (**d**–**f**) for *y* directions.

In applying the CWT based frequency inversion method, only the recording of the sensor at the roof location is used. The method originally detects the frequency of vibration by using the information present at the tail. This choice eliminates the forced vibration and ends up with the natural vibration frequency. In Figure 10, an example inversion is made for the TURN building during the 25 May 2021 event, where time–acceleration and time–frequency–acceleration amplitudes are illustrated in addition to the corresponding EFDs through the use of tail information (e.g., last 10 s) of the recorded EGMs.

**Figure 11:**
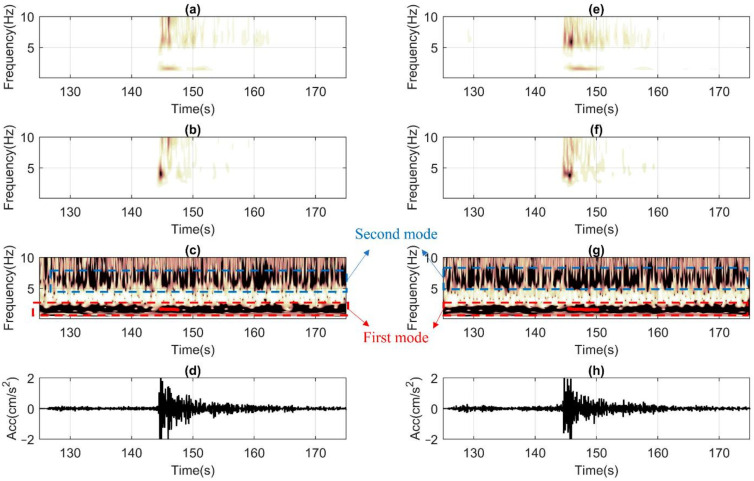
Post-processing the 25 May seismic response of the TURN building, which is monitored through high precision TSA-100S sensors. (**a**,**e**) ST of the roof signal for *x* and *y* horizontal components, (**b**,**f**) similar to a and e but the base signal, (**c**,**g**) ST-Amp functions with the automatic identification of the natural frequencies during the strong shaking along with *x* and *y* directions, (**d**,**h**) recorded acceleration time histories along *x* and *y* at the base level.

Figure 11 presents an ST-AF based elaboration of recorded ground motion data belonging to the 25 May event acquired by the high precision sensors installed on the TURN building in terms of (a and e) time (*t*)-frequency (*f*) response at the roof, (b and f) *t-f* response at base, (c and g) *t-f* amplification, and (d and h) acceleration (*acc*)—*t* history at the base. This case constitutes an ideal example, where fundamental vibration frequencies of about *f_0,H1_* = 1.6 Hz and *f_0,H2_* = 1.6 Hz (for both horizontal directions) could be sampled independently from the temporal domain of analyses (i.e., noise: *t* < 120 s, weak motion: 120 s ≤ *t* ≤ 130 s, or tail: *t* > 130 s). Moreover, the data clearly shows the activation of the second structural horizontal mode with frequencies of about 6–7 Hz, as well. From the ST of the ground motions (i.e., b and f), it could be appreciated that the source has generated frequencies larger than 3–4 Hz, which are possibly amplified by the higher modes of the soft and deep soil deposit lying below [89,90]. The propagation of which is also visible at the roof responses (i.e., a and e). Finally, high signal/noise ratios are evident from the absence of high-intensity information in the noise domain.

### 5.2. Comparison of the Methods Considering the Recordings from the RS4D Sensors Installed in the LAS Building

With a compromise in the assumption that the input motion can be represented as a white noise, FDD is performed to view the singular values of multi-channel data from the RS4D device arrays as well as the high-fidelity instrumentation. It is expected that the singular values from the high-fidelity instruments possess and reflect high signal-to-noise ratio, whereas the spectral features from RS4D are strongly affected by the signal noise. As a result, the spectral features pointed out from RS4D are expected to be less significant, in other words, the spectral peaks are more difficult to identify. However, the feasibility depends on the level of vibration and low-amplitude events may not generate sufficient vibration to support identification processes via RS4Ds.

**Figure 12:**
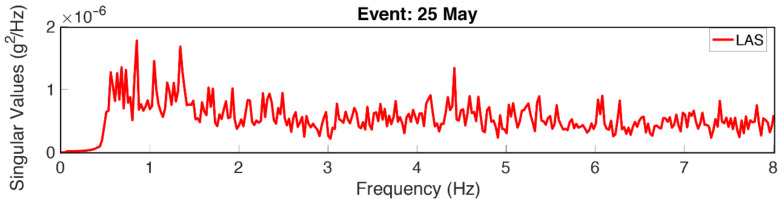
SVD as the output of FDD along the *x*-direction through the use of the dataset of the 25 May event recorded on LAS.

In this section, the focus is made on underlining the negative effects of low S/N ratios present in RS4D sensors on the operations of frequency identification methods and workaround solutions employed to enhance the identification procedure. An illustration is made by considering the LAS building response under the 25 May event. To start with, as shown in Figure 12, the SVD of FDD is presented. As it is observed, instrumental self-noise shows itself in terms of multi peaks with respect to the relatively clear response obtained in the previous section. Identification of the natural frequency of vibration may be achieved through the visualization of the structural modal shapes and repeatability analyses through applying different input signals. From the modal shape analyses, the LAS building frequency is identified as about 1.3 Hz.

**Figure 13:**
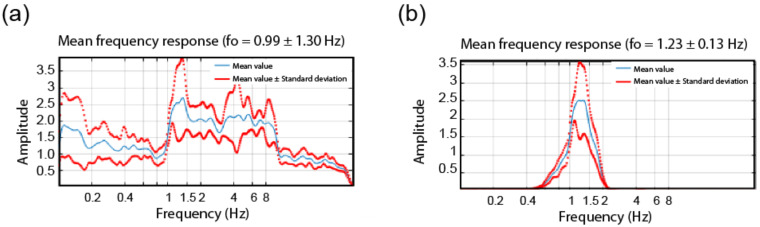
FAD as the output of STFT-WF of LAS building along the *x*-direction, (**a**) without the use of wavelet pre-filtering, (**b**) with the use of wavelet pre-filtering.

In the case of STFT-WF, low values of S/N ratios impose the use of a wavelet pre-filter, the frequency band of which is predetermined. In Figure 13, identification results of the same building are shown for x-direction with and without the use of the wavelet pre–filtering option. It can be observed that FAD with pre-filtering provides a precise identification of the building’s fundamental frequency of about 1.23 Hz.

**Figure 14:**
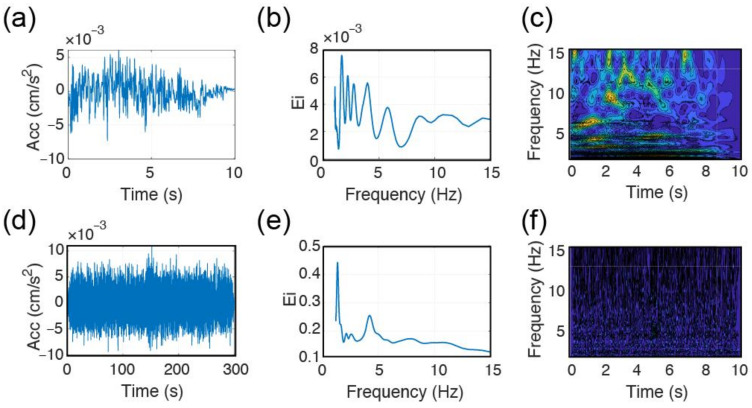
CWT based processing of LAS response along the x-direction, (**a**) acc-t history of the tail, (**b**) EFD of (**a**), (**c**) t-f representation of (**a**), (**d**) acc-t history of the ground motion, (**e**) EFD of (**d**), (**f**) t-f representation of (**d**).

When it comes to CWT-based elaboration, digital noise creates troubles in the identification of the building vibration in the tail domain. Having the tail response not clearly identifiable, EFD becomes polluted by spurious peaks (Figure 14a–c). This limitation could only be overcome by forcing the approach to operate in the entire domain instead of tail-only. Such enforcement will also bring some liabilities with it, like the possibility of identification of the input frequencies alongside the natural ones. Although this would be true in general terms, given the limited frequency information present in the source (*f* > 3–4 Hz) and the likelihood of having *f*_0_ > 3 Hz is rather limited for the dataset under consideration apart from BAL. Hence, the main assumption that is being made is that the building response will be dominated by its first natural vibration frequency independently from the input motion. Although this assumption is important, it is underlined that a stronger event will provide also more noise-free tail information, which will enable to return to the use of tail-only information. In Figure 14d–f, following the inversions made by using the entire ground motion domains, natural frequency of vibration for LAS could be successfully finalized as *f*_0,*x*_ of about 1.35 Hz.

**Figure 15:**
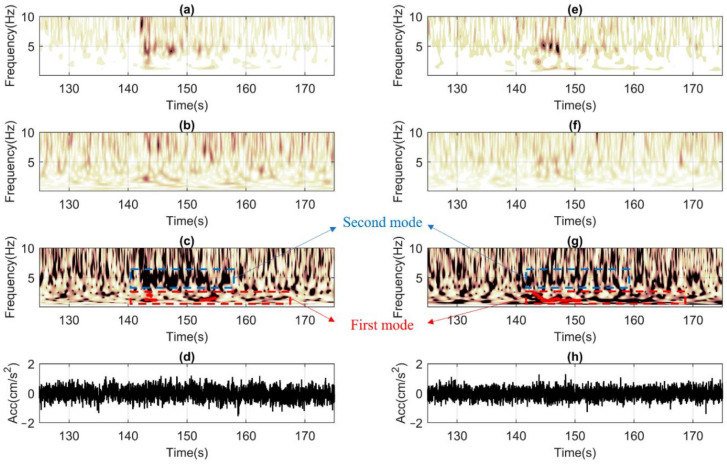
Post-processing the 25 May seismic response of the LAS building, which is monitored with the low-cost RS4D sensors. (**a**,**e**) ST of the roof signal for *x* and *y* horizontal components, (**b**,**f**) similar to a and e but the base signal, (**c**,**g**) ST-Amp functions with the automatic identification of the natural frequencies during the strong shaking along with *x* and *y* directions, (**d**,**h**) recorded acceleration time histories along *x* and *y* at the base level.

Finally, in Figure 15, ST-AF based processing is shown. It could be observed that the post-processing becomes less accurate before the ground motion arrives. It is noted that high amplitudes of digital noise are more concentrated with f > 2 Hz. Unfortunately, the random-natured temporal distribution of the recorded noise renders it impossible to rely on solely noise-based measurements. The natural vibration frequency could only be captured once the structure starts oscillating during the weak motion with around f_0,x_ = f_0,y_ = 1.4 Hz. The second vibration mode frequencies are less clear, but still recognizable at about 4 and 5 Hz. Once the weak motion finishes, the noise level pollution re-enters and makes the inversion becomes slightly more unstable. Yet, despite all the limitations listed above, the method still could be acceptable for SHM purposes.

### 5.3. Method Repeatability in Terms of Frequency Inversions Considering DRG and BAL

**Figure 16:**
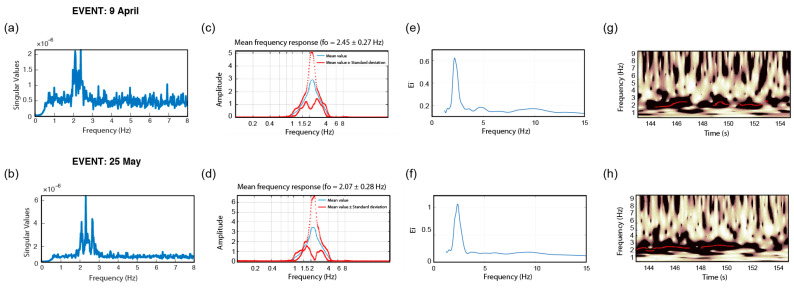
Repeatability analyses of frequency inversion considering x-direction of the DRG response in terms of (**a**,**b**) SVD of FDD, (**c**,**d**) FAD of STFT-WF, (**e**,**f**) EFD of CWT, and (**g**,**h**) SAD of ST-AF, (**a**,**c**,**e**,**g**) show the results for 09 April 2021 and (**b**,**d**,**f**,**h**) show the results for 25 May 2021.

**Figure 17:**
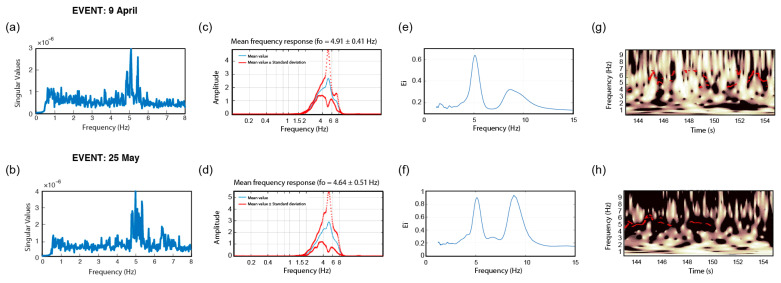
Repeatability analyses of frequency inversion considering x-direction of the BAL response in terms of (**a**,**b**) SVD of FDD, (**c**,**d**) FAD of STFT-WF, (**e**,**f**) EFD of CWT, and (**g**,**h**) SAD of ST-AF, (**a**,**c**,**e**,**g**) show the results for 9 April 2021 and (**b**,**d**,**f**,**h**) show the results for 25 May 2021.

In this section, method repeatability is illustrated for two sample buildings, DRG and BAL, by comparing the final output of the modal identification approaches (along the x-direction) considering the two events recorded on 9 April and 25 May 2021. It should be noted that due to the reasons explained in the previous section, wavelet pre–filtering is used for STFT-WF and EGM domain is used for FDD and CWT approaches. The relevant comparisons are shown for DRG in Figure 16 and for BAL in Figure 17.

It could be observed from Figure 16 that, considering the response of the DRG along the x-direction, a precision loss is noted for the approaches of FDD, STFT-WF, and ST-AF, whereas the results of CWT show a superior consistency. Nevertheless, the precision loss is not found striking in general terms. Hence, despite the presence of low S/N ratios, the stability of the inversions is concluded to be convincing enough. On the other hand, when it comes to the response of the BAL building (shown in Figure 17), especially the ST-AF method did not work well in the case with lower S/N ratios (i.e., see (f) for the corresponding inversion). Despite the higher variations with respect to DRG, the other approaches performed better as compared to ST-AF. This comparison can be taken as a benchmark for cases where the high noise affects the most postprocessing with ST-AF, as being the only one in terms of an input–output approach with noisy input.

Table 4 reports the fundamental frequencies obtained considering the four modal identification approaches for the entire set of instrumented buildings, together with the mean values and coefficient of variation (CoV). It could be commented from the presented results that for the less noisy configuration (e.g., TURN) all SHM approaches are able to provide very similar fundamental frequency estimates. Among the two events of the 9 April and 25 May, the former resulted in lower S/N ratios. When the focus is given to this event, it could be observed that there is a significant scatter in the results (i.e., high CoV values), particularly in the Y direction. Among the methods, the predictions of STFT-WF, CWT, and FDD are influenced less by high noise concerning ST-AF which showed scattering of the results of about 40% (i.e., the case of LAS). When the level of S/N increases, all the methods provided more consistent estimations. Among all SHM approaches, ST-AF becomes the best performer with the maximum deviation from the mean with only about 10% (i.e., the case of DRG). Overall, the SHM approach associated with the smallest deviations from the mean is the one based on CWT. Finally, it could be concluded that all of the SHM approaches are expected to perform better in case of a larger magnitude event which would impose greater acceleration demands.

### 5.4. Comments on the Future Usability of the RS4D Sensors for Rapid Response to Earthquakes (RRE) Purposes

As it has been discussed up to now, the weak nature of the recorded motions and high self-noise levels of the RS4D sensors are posing some limitations in the signal processing phase. Yet, these limitations may be overcome with a dataset belonging to a stronger event that generates motions with a higher signal to noise ratios rendering the use of RS4Ds suitable for the application in terms of RRE in Bucharest.

If one considers a 5.0 magnitude event with a similar epicenter as the Vrancea 1977 earthquake (160 km NE of Bucharest), occurring at 150 km depth, the expected mean peak ground acceleration (PGA) will reach 4.0–4.5 cm/s^2^ in the city of Bucharest, according to the regional ground motion model (GMM) of Vacareanu et al. [91]. When the same GMM is used with the depth of the hypocenter of 90 km, the expected value of PGA further rises to 6.0–6.5 cm/s^2^. For PGAs greater than 4 cm/s^2^, the intense part of the ground motions recorded by RS4Ds would be even visually recognisable sensors since signal levels lie above the digital self-noise level of about 1 cm/s^2^. When one considers the expected damage output, these levels of PGAs are not expected to cause any damage. This can be concluded based on the fragility functions defined in [92], corresponding to the structure with the longest fundamental period of vibration among those considered in this study (i.e., LAS, 0.7–0.8 s), more sensitive to strong long-period ground motion in Bucharest [93].

One of the overall ambitions of the TURNkey project is to provide an efficient procedure for rapid prediction of losses immediately following an earthquake. The added value of the RS4D sensors data will be of critical importance in TB1, Bucharest. For future rapid damage assessment purposes in TB1, in case of an event with M > 5.0, the proposed approach for damage assessment will be the empirical method presented in [16], which correlates with the reduction in the fundamental frequency to EMS98 damage class predictions.

## 6. Concluding Remarks

This work investigates for the first time the usability of RS4D low-cost sensors for dynamic identification of structures. The database analyzed includes the existing buildings in Bucharest, Romania consisting of 4 reinforced concrete (RC) and 1 masonry building monitored with RS4D sensors plus an additional RC building equipped with high-precision TSA-100S sensors. SHM techniques for structural identification are FDD, STFT-WF, CWT, and ST-AF. By using the data obtained during two recent low magnitude events of 9 April 2021 and 25 May 2021, the following conclusions are obtained:

- Self-noise of RS4D sensors is too high to rely on noise-based SHM methods. For this reason, the domain of FDD is set as EGM and during structural identification, continuous verifications of deformed shape and mode repeatability are consistently confirmed.
- Low S/N ratios present in RS4D sensors adversely affect the precision in terms of frequency identification, the noisier the signal becomes the less becomes the precision of the inversions.
- Unlike standard SHM configurations accessing ambient vibration data, useful measurement lengths with RS4D are short due to the earthquake durations limiting the quality of identification results. This limitation becomes even more prominent when subsets of the measurements are of concern (e.g., tail data).
- Following up from the above, utilization of output-only techniques possesses risks due to the dominance of input signal content with particular spectral characteristics. With low S/N ratios and short-duration measurement issues expressed above, this concern becomes more influential.
- Among the methods, the most precise SHM approach in terms of deviations from the mean is found to be CWT. Besides, executing multiple identification techniques in parallel can increase the confidence level in terms of identification accuracy.
- All SHM techniques provided precise identification for the control building monitored with TSA-100S sensors. This conclusion proves that with high S/N ratios, all methods work reliably.
- It is expected that for a damaging event in Bucharest, RS4D sensors will operate sufficiently well to record the changes in the oscillation periods, which will be correlated with structural damage defined in [16].

In summary, RS4D platforms are promising in the way they can deliver structural features if vibration levels are sufficient to overcome the high noise levels. In seismically active regions, sufficient vibration levels can be acquired depending on the shaking intensity, yet with the limitations expressed above. Nevertheless, as the RS4D network grows larger, it is indispensable that they will find optimal formulations serving as alternative SHM portals with minimal cost and labor requirements, which are the driving forces of this technology in the intersecting areas of engineering seismology and SHM. Europe-wide implementation of RS4Ds through TURNkey undoubtfully supports this vision and is expected to produce a broader dataset contributing to this cause in the near future.

## Figures and Tables

**Table 1:** Main characteristics of the instrumented structures within the TURNkey project. A: Attic, B: Basement, GF: Ground floor, S: Story.

Building Code	Construction Year	Structure Type	Building Configuration	Sensors Installed	Location of the Sensors
DRG	1982	Large panel structure (precast shear walls structure)	B + GF + 8S	3 RS4D	B, 5th S, 8th S
BAL	before 1963 (<1940)	Unreinforced Masonry	B + GF + A	2 RS4D	B, A
DRT	before 1963	Large panel structure (precast shear walls structure)	GF + 8S	3 RS4D	GF, 5th S, 9th S
TIT	1963–1977	Large panel structure (precast shear walls structure)	B + GF + 10S	3 RS4D	GF, 5th S, 10th S
LAS	2008	Reinforced Concrete frame	3B + GF + 11S	4 RS4D	3rd B, GF, 5th S, 11th S
TURN	1973 (retrofitted in the 1990s)	Reinforced Concrete shear walls	B + GF + 9S	3TSA-100S	B, 6th S, 10th S

**Table 2:** Earthquakes that occurred in Romania after the deployment of the TURNkey instruments (June 2020) which covers one year, with M_L_ ≥ 3.8. Selected events are highlighted in blue.

EQ NO	DATE	TIME (UTC)	DEPTH (km)	M_L_
1	2 June 2020	11:12:58	101.3	4.5
2	21 June 2020	03:47:27	119.4	4
3	25 June 2020	17:30:16	12.4	4.5
4	15 August 2020	02:28:54	125.1	3.9
5	10 October 2020	06:29:47	65.8	4
6	22 October 2020	20:21:44	122.1	4
7	29 October 2020	22:39:37	9.1	4.2
8	3 November 2020	09:14:41	117.7	3.9
9	27 November 2020	12:30:16	124.9	3.8
10	23 December 2020	14:27:17	126.2	3.8
11	5 January 2021	23:50:34	9.6	4
12	14 February 2021	17:24:52	126.2	3.8
13	24 February 2021	02:35:09	133.9	4
14	27 February 2021	21:13:11	124.8	4.2
15	7 March 2021	09:52:28	144.5	4.1
16	7 March 2021	22:34:28	144.9	3.9
17	23 March 2021	06:26:54	121.3	3.8
**18**	**9 April 2021**	**18:36:47**	**79**	**4.6**
19	18 April 2021	10:03:16	134.7	3.8
**20**	**25 May 2021**	**21:30:37**	**131.2**	**4.7**

**Table 3:** Summary of the SHM techniques adopted in the TURNkey project.

SHM Technique Utilized	Acronym	Approach	Intermediary Output
Frequency domain decomposition	FDD	Output only	Singular Value Diagram (SVD)
Short-time Fourier transform with wavelet pre–filtering	STFT-WF	Output only	Fourier Amplitude Diagram (FAD)
Continuous wavelet transform	CWT	Output only	Energy-Frequency Diagram (EFD)
Stockwell transformation-amplification function	ST-AF	Input-output	Stockwell Amplification Diagram (SAD)

**Table 4:** Summary of the fundamental frequencies identified through several different modal identification approaches. CoV is the coefficient of variation of the frequency identifications carried out by using different approaches. Question mark superscript (^?^) indicates that the frequency identification outcome is not precise (see Figure 17f, for an example imprecise result). For the calculation of CoV, the standard deviation is computed based on the population.

Building Sensor	f_0_ (Hz) Dir.	FDD 09/04|25/05	STFT-WF 09/04|25/05	CWT 09/04|25/05	ST-AF 09/04|25/05	Mean 09/04|25/05	CoV 09/04|25/05
BAL	*x*	5.10|5.03	4.91|4.64	4.92|5.00	5.7 ^?^|5.2	5.16|4.97	0.06|0.04
RS4D	*y*	3.71|6.59	4.59|5.18	5.56|6.25	5.5 ^?^|5.8	4.84|5.96	0.16|0.09
DRG	*x*	2.42|2.32	2.45|2.07	2.21|2.17	2.4|2.5	2.37|2.27	0.04|0.07
RS4D	*y*	3.05|3.25	2.40|2.19	3.00|3.48	2.9|3.1	2.84|3.01	0.09|0.16
DRT	*x*	2.91|2.69	2.23|2.04	2.77 ^?^| 2.77 ^?^	2.7|2.7	2.65|2.55	0.10|0.12
RS4D	*y*	1.88|1.91	2.10|2.28	1.94|1.85	2.3 ^?^|1.9	2.05|1.99	0.08|0.09
LAS	*x*	1.37|1.34	1.30|1.23	1.36|1.33	1.4|1.4	1.36|1.33	0.03|0.05
RS4D	*y*	1.20|1.20	1.21|1.20	1.20|1.23	2.1 ^?^|1.4	1.42|1.25	0.27|0.07
TIT	*x*	1.88|1.98	1.96|2.04	1.86|1.96	2.1|2.1	1.95|2.02	0.05|0.05
RS4D	*y*	2.42|2.37	2.10|2.28	2.38|2.36	2.4|2.4	2.33|2.35	0.06|0.02
TURN	*x*	1.56|1.59	1.55|1.58	1.54|1.54	1.6|1.6	1.56|1.58	0.01|0.01
TSA-100S	*y*	1.56|1.56	1.54|1.58	1.46|1.52	1.6|1.6	1.54|1.57	0.03|0.02

## Data Availability

All data included in the manuscript are available upon request by contacting the corresponding author.
